# RURAL HEALTH, FAMILY CAREGIVING, AND DEMENTIA: CHARACTERISTICS AND NEEDS ACROSS POPULATIONS

**DOI:** 10.1093/geroni/igad104.1706

**Published:** 2023-12-21

**Authors:** Caroline Stephens, Rebecca Goodwin, Katherine Ornstein

## Abstract

The rural population in the US is aging faster, more likely to live alone, and has more comorbidities than the overall aging population. Despite the greater need for long-term care services, rural residents may experience the most barriers to formal services (hospital, home health) and availability of family caregivers. Unfortunately, there is a dearth of research on aging and end-of-life care in rural areas. This symposium includes 4 presentations focused on rural health, family caregiving and dementia and the characteristics and needs across these diverse populations. The first presentation examines rural disparities in use of family and formal caregiving for older adults with disabilities using data from the Health and Retirement Study. The second presentation describes the unmet services needs among people with dementia and their caregivers living in rural Appalachia, and highlights contextual factors that shape service access and utilization. The last two presentations use data from the Utah Population Caregiving Database to examine the characteristics of families of nursing home (NH) residents who die with dementia and of those who die in rural NHs. Understanding the unique characteristics and needs of these underserved populations will help inform rural health practice and policy considerations to better serve this population, especially as we continue to see changes in the hospital and NH landscape across the US. Moreover, it is critical that even as federal and state policies rebalance long-term care from institutional settings to home- and community-based settings, the unique needs of those living in more rural areas are considered.

